# A call for action in bleeding prevention

**DOI:** 10.18632/aging.101745

**Published:** 2019-01-11

**Authors:** Felice Gragnano, Negar Manavifar, Marco Valgimigli

**Affiliations:** 1 Department of Cardiology, Bern University Hospital, University of Bern, 3010 Bern, Switzerland

**Keywords:** acute coronary syndromes, percutaneous coronary intervention, bivalirudin, radial access

Preventing ischemic complications while limiting bleeding risk is the cornerstone when treating patients with acute coronary syndrome (ACS) undergoing percutaneous coronary intervention (PCI). Questions still persist regarding the best strategies for minimizing the risk of bleeding events when considering their actual negative impact on cardiovascular outcomes and mortality [1]. As is often the case, these questions become even more challenging in the frail and elderly population. During the last decade, there have been heated debates over the preferred arterial access (radial versus femoral), and antithrombotic regimen (bivalirudin versus unfractionated heparin) which are the major factors blamed as being responsible for bleeding complications.

The MATRIX (Minimizing Adverse Haemorrhagic Events by Transradial Access Site and Systemic Implementation of Angiox) trial sought to solve this dispute [2,3]. The MATRIX was a research programme of three nested randomized multicentre open-label trials enrolling 8,404 patients with an ACS for whom PCI was planned (elderly patients aged ≥75 years accounted for approximately 25% of the population) [2–4]. The three main purposes of the MATRIX investigators were to compare the safety and eﬀectiveness of a) radial versus femoral access (MATRIX Access); b) bivalirudin versus heparin (with optional glycoprotein IIb/IIIa inhibitors [GPI]) (MATRIX Antithrombin); and c) post-PCI bivalirudin infusion versus no post-PCI infusion (MATRIX Treatment Duration). Among patients assigned to post-PCI bivalirudin infusion, the dose could have been either full or reduced at the discretion of the treating physician.

The co-primary endpoints selected for the MATRIX Access and the MATRIX Antithrombin were the occurrence of major adverse cardiovascular events (MACE) (defined as a composite of death, myocardial infarction, or stroke), and net adverse clinical events (NACE) (a composite of major bleedings [Bleeding Academic Research Consortium (BARC) type 3-5] or a MACE) up to 30-days. The primary endpoint for MATRIX Treatment Duration was a composite of urgent target-vessel revascularization, definite stent thrombosis, or NACE [2–4].

The primary outcomes at 30-days follow-up of the three nested trials were published in 2015. The MATRIX Access showed that the use of radial access compared to femoral access was of benefit with respect to NACE (9.8% vs. 11.7%, rate ratio [RR] 0.83, 95% confidence interval [CI] 0.73-0.96; p=0.0092), but not MACE (8.8% vs. 10.3%, RR 0.85, 95% CI 0.74-0.99; p=0.0307, non-significant at α of 0.025). The reduction in NACE was driven by major bleeding unrelated to coronary artery bypass graft surgery and all-cause mortality [2]. In the MATRIX Antithrombin the 30-days comparison between bivalirudin and heparin showed neutral findings, not significantly reducing the rate of MACE (10.3% and 10.9%; RR, 0.94; 95% CI, 0.81-1.09; p=0.44) nor NACE (11.2% and 12.4%; RR, 0.89; 95% CI, 0.78-1.03; p=0.12). Similarly, the primary composite outcome did not differ between patients with post-procedural bivalirudin infusion as compared to with no infusion (11.0% and 11.9%; RR, 0.91; 95% CI, 0.74-1.11; p=0.34) [3].

Recently, these findings have been reinforced by the publication of the prespecified final 1-year analysis of the whole MATRIX programme, confirming the previously reported 30-days outcomes in all respects. In the MATRIX Access, results showed a carry-over benefit for radial access compared to femoral access with respect to NACE at 1-year (15.2% vs. 17.2%; RR 0.87, 95% CI, 0.78-0.97; p=0.0128) mainly driven by a reduction in major bleeding events and cardiovascular death. For MACE, a trend for reduction was observed but remained formally nonsignificant (14.2% vs. 15.7%; RR 0.89, 95% CI 0.80-1.00; p=0.0526) [4]. In the MATRIX Antithrombin, bivalirudin did not significantly reduce MACE (15.8% vs. 16.8%; RR 0.94, 95% CI, 0.83-1.05; p=0.28) NACE (17.0% vs 18.4%; RR 0.91, 95% CI 0.81-1.02; p=0.10) as compared with heparin. Besides, post-PCI bivalirudin infusion did not significantly lower the rate of the 1-year primary endpoint compared with the no-infusion strategy (17.4% vs. 17.4%; RR 0.99, 95% 0.84-1.16; p=0.90) [4].

Results from the MATRIX programme argue in clear favor of radial over femoral access for reducing adverse clinical events in patients with ACS undergoing invasive management [2,4,5] and have been firmly incorporated into the European Guidelines that recommended radial artery as the preferred vascular access site for any PCI [6,7].

On the other hand, the comparison between the two antithrombin strategies remains difficult to interpret [3,4,8]. Although the MATRIX Antithrombin failed to show a significant superiority over bivalirudin with respect to NACE or MACE, intriguing messages emerged from secondary analyses. Firstly, at both 30-days and 1-year follow-up, bivalirudin was associated with a lower rate of all-cause and cardiovascular mortality, and bleeding complications [2,4]. Secondly, the use of bivalirudin was associated with a reduction in both access site and non-access site-related bleeding events as compared with heparin [3,4,8], irrespective of the use of planned GPI [8]. Thirdly, although a slight increase in ischemic events including stent thrombosis was recognized using bivalirudin, administration of full-dose post-PCI infusion instead of low-dose mitigated the risk of thrombotic complications. Finally, at 1-year follow-up there were trends for both co-primary endpoints favoring the use of bivalirudin in patients with impaired renal function, generally identifying an older and more frail population. Hence generating the hypothesis that this treatment might be advantageous in this special population [4]. All these considerations keep a door open for a bivalirudin-based strategy during PCI and warrant further investigations.

Thus, the MATRIX programme provided prospective findings, arising from a large and multinational investigation, supporting a cause-effect relationship between bleeding mitigation strategies and mortality benefit. Considering that advanced age per se is amongst the most important bleeding risk features [7], every effort should be made particularly in elderly patients to apply the new gold standards for bleeding prevention in current practice.
